# Novel risk index integrating practical operation limits enhances probabilistic contingency ranking for large-scale photovoltaic plant planning

**DOI:** 10.1038/s41598-024-60024-7

**Published:** 2024-05-01

**Authors:** Rasha Elazab, Mohamed K. El-Aser, Adel A. El-samahy

**Affiliations:** Electrical Power and Machines Department, Faculty of Engineering, Helwan University, Cairo, Egypt

**Keywords:** Contingency analysis, Power system planning, Probabilistic modelling, Photovoltaic, Risk assessment, Electrical and electronic engineering, Photovoltaics

## Abstract

This research addresses the pressing need for heightened grid security amid increasing uncertainties in photovoltaic PV generation. The research problem lies in the limitations of conventional contingency analysis metrics, failing to adequately consider both contingency occurrences and uncertainties inherent in PV generation. In response, a comprehensive algorithm is proposed that introduces a novel severity function framework, enhancing traditional contingency ranking metrics. This approach incorporates grid remedial actions and refines line and bus voltage classification by considering available correction time, aiming to offer a more robust security assessment. Motivated by the imperative to address uncertainty in PV generation, the proposed work builds on established analysis tools. A probabilistic load flow algorithm manages PV generation uncertainties, utilizing historical data for contingency incidence uncertainty. Additionally, a probabilistic model for PV plants integrates historical solar data, deriving hourly probability density functions to meet grid code requirements, including reactive power considerations. The justification for this work lies in the algorithm's demonstrated efficacy, validated on the IEEE 14-bus network. Results highlight its ability to identify risks associated with line overloading and bus voltage breaches. Comparative evaluations underscore proper coupling buses for security, favoring distributed capacity to mitigate line overloading risks. The study's key results emphasize voltage risk amplification with reactive power omission, stressing the significance of compensation strategies. This research addresses a critical problem, presenting a comprehensive algorithmic solution to enhance grid security amidst uncertainties in PV integration. Findings offer valuable insights for strategically interaction between large scale PV plants and electrical grid, contributing to an improved grid security paradigm in a dynamic and uncertain energy model.

## Introduction

The persistent evolution of the electrical power network, driven by the rising demand for electricity, is unmistakably evident. The sophisticated interconnections within the power grid make it susceptible to cascade events, where a failure in one segment triggers a ripple effect through interconnected components. These cascading events, often initiated by the disconnection of a single transmission line, have the potential to lead to widespread line failures and extensive blackouts. The severity of such cascading failures is assessed based on factors such as the count of tripped lines, the duration of the event, and the magnitude of load shedding required for restoration^1^.

N-1 contingency analysis is a practice that involves evaluating the network's resilience by simulating the removal or malfunction of a critical component, such as a transmission line. These analyses provide insights into potential vulnerabilities that could catalyze cascading failures and blackouts, serving as a foundation for informed decision-making to enhance grid reliability and minimize the repercussions of unforeseen incidents^2,3^.

The assessment and ranking of possible contingencies serve as tools to identify optimal corrective and remedial strategies for improving grid vulnerabilities^4,5^. The development of ranking methodologies dates back over five decades, encompassing various performance metrics proposed in the existing literature^6,7^. Additionally, composite indices, such as the voltage-reactive performance index^8^, and amalgamated metrics like the composite security index or combined performance index^9^, have been introduced. A recent contribution^10^ introduces a hybrid index for assessing bus voltage, incorporating factors such as active power and voltage angle to enhance contingency ranking precision.

Furthermore, the Forced Outage Rate (FOR), calculated by integrating historical grid data, predicts the probabilities of requisite events^11,12^. Risk functions, like conventional performance indices, prioritize contingencies without considering grid remedial actions. Artificial intelligence methods^13,14^, rooted in the foundational principle of performance indices, have emerged for contingency ranking. Notable techniques include fuzzy logic^15^, support vector machines^16,17^, and artificial neural networks^18,19^.

However, existing experiences in contingency ranking reveal substantial deviations of individual grid components by applying high-powered ranking indices that moderate relatively minor distributed deviations. Current ranking indices often overlook the temporal aspect of protective actions and the potential for cascading outages triggered by numerous minor deviations in multiple grid zones.

Moreover, the management of expansive photovoltaic (PV) installations introduces notable complexities regarding the security and dependability of power systems. Extensive research has focused on utilizing contingency analysis as a foundational tool to assess the secure integration of PV sources into local grids. Case studies from diverse grids, such as Cameron^20^, Malaysia^21,22^, Cyprus^23^, and Nigeria^24^, provide valuable insights.

PV facilities are inherently exposed to various uncertainties, including fluctuations in solar irradiance, prevailing weather conditions, and grid disturbances, impacting their power output and voltage profiles. The operational feasibility of PV plants is constrained by practical thresholds, including power factor considerations, voltage regulation imperatives, and reactive power provisioning.

Probabilistic contingency analysis plays a pivotal role in accounting for uncertainties in power system components, departing from classical contingency analysis^25^. By employing sophisticated probabilistic models and methodologies, this approach facilitates evaluating the probability and implications of diverse scenarios, including solitary or multiple contingencies, load fluctuations, and variations in renewable energy generation^26,27^.

Numerous studies have refined various probabilistic techniques and models tailored to power system contingency analysis, including rare event approximation, cut-set probability truncation, binary decision diagrams, and the application of Markov models^28,29^.

In power system research, various probabilistic PV models aim to replicate PV power patterns using a singular probability density function (PDF). However, these models often neglect the impacts stemming from temperature-related uncertainties and the reactive power support associated with PV systems.

A comprehensive approach in^30^ concurrently considers uncertainties in both temperature and solar radiation through the utilization of an optimized PDF, revealing distinct hourly fitting PDFs for solar radiation across Normal, Beta, and Weibull distributions. Reactive power support is modeled to provide a complete understanding of PV behavior, emphasizing the importance of evaluating the consequences of curtailing PV plant integration for upholding power system stability.

In summary, the principal contributions of this research can be outlined as follows:

- Introduction of a novel approach incorporating severity functions, considering potential grid remedial actions, resulting in marked enhancements in contingency ranking compared to conventional indices.
- Presentation of a probabilistic contingency analysis framework that encompasses contingencies and inherent variability in PV generation, along with operational guidelines applicable to both conventional and PV generation contexts for line loading and bus voltages.
- Application of a model reliant on historical solar radiation and temperature data spanning multiple years, producing hourly PDFs for both factors, and consideration of diverse grid code requirements governing PV plant interconnection to establish reactive power prerequisites.

**Figure 1:**
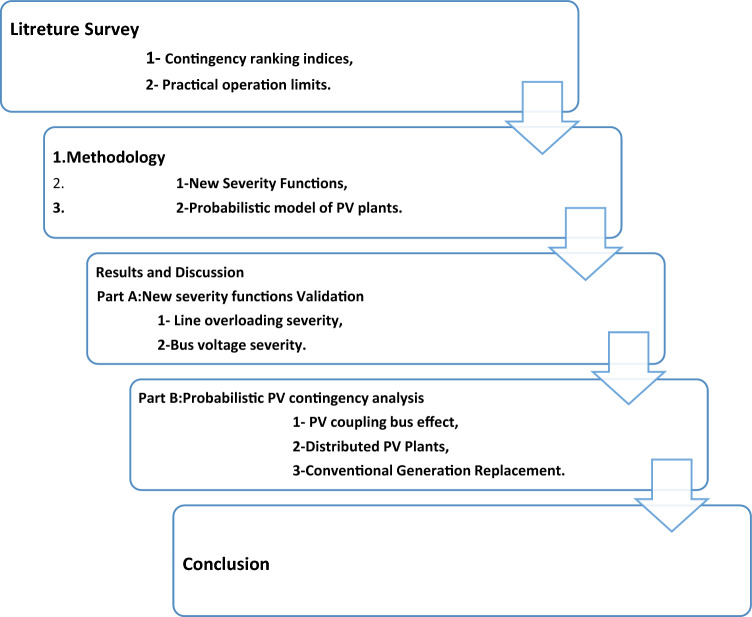
Paper graphical framework.

The structural framework of the paper encompasses a thorough literature survey, wherein relevant experiences with existing contingency indices are examined, and operational limits of the grid are briefly outlined in Section "Literature survey". Section "Methodology" introduces novel severity indices along with a comprehensive probabilistic model of PV plants. The outcomes of the study are deliberated in Section "Results and discussion". The main conclusions derived from this study are underscored in Section "Conclusion", as illustrated in Fig. 1.

## Literature survey

### Contingency ranking indices

The conventional contingency ranking metrics can be categorized into two distinct classes: performance indices and risk indices. These classifications will be further clarified in the subsequent paragraphs.

#### Performance indices PIs

Performance Indices PIs, that represent deviations in voltages and occurrences of line overloads find common applications. These indices are derived through meticulous post-contingency power flow analyses. Contingencies that lead to violations of system constraints—such as load bus voltages exceeding their established normal limits or instances of transmission line overloads—result in a notable escalation of PI values. It is noteworthy that an array of diverse PI formulations has been conceived and implemented, contributing to the spectrum of tools available for effective contingency ranking endeavors. The most common form of performance indices can be found as^31^:

1 $$F = \sum w_{i} *\left( {\frac{{x_{i} }}{{X_{i} }}} \right)^{2n}$$

where *F*: The performance index, *W*_*i*_: The weight factor, *x*_*i*_: The value of post-contingency system variable, *X*_*i*_: The upper limit of variable, $n$: a positive integer.

The phenomenon of masking effect arises due to the presence of the exponent 2n. When a relatively small value is assigned to 2n, it results in heightened challenges in precisely distinguishing between scenarios and contingencies of severe and non-severe operational conditions^5^. Conversely, opting for a larger value of 2n mitigates the masking effect; however, this choice introduces computational intricacies stemming from the augmented number of multiplicative operations^32^.

#### Risk indices

This metric combines considerations of limit violations with the failure rate of various system elements^13^. It accounts for both the probability of event occurrences and the resulting consequences associated with those events. To estimate the FOR, historical data from the grid is utilized, facilitating the prediction of probabilities for potential events.

2 $$\mathbf{R}\mathbf{i}\mathbf{s}\mathbf{k}\left(\mathbf{S}\mathbf{C}\right)=\sum \mathbf{P}\mathbf{r}\left({\mathbf{C}}_{\mathbf{i}}\right)\mathbf{*}\mathbf{S}\mathbf{e}\mathbf{v}\left({\mathbf{C}}_{\mathbf{i}},\mathbf{S}\mathbf{C}\right)$$

Where, *C*_*i*_: *i*-th contingency. SC: The system condition (demand, generation), Pr(*C*_*i*_): The probability of contingency *C*_*i*_,, Sev(*C*_*i*_, SC): The severity of *i*-th contingency under SC loading condition, it reflects post-contingency line overloading and bus over/under voltage violations.

According to^12^ severity is quantified with the same previous power and voltage performance indices.

Therefore, the risk index is calculated as:

3 $$RI_{v} = \mathop \sum \limits_{i = 1}^{NB} Pc*W_{i}^{v} *\left( {\frac{{V_{i} - V_{i}^{setpoint} }}{{\Delta V^{max} }}} \right)^{2n}$$

4 $$RI_{S} = \mathop \sum \limits_{i = 1}^{NL} Pc*W_{i}^{s} *\left( {\frac{{S_{i} }}{{S_{i}^{max} }}} \right)^{2n}$$

where ${RI}_{v}$: Risk of bus over/under voltage, ${RI}_{S}$: Risk of line overloading, *Pc*: Probability of contingency, *W*_*i*_: weighting factor according to bus/line importance in the grid.

Line overloading and bus voltage severity functions are introduced in^13^ respectively as:

5 $${\text{Volt Sev}}\left( {V_{j} } \right) = \left\{ {\begin{array}{*{20}l} {\frac{{0.94 - V_{j} }}{0.94},} \hfill & {\left| {V_{j} } \right| < 0.94} \hfill \\ {0,} \hfill & {0.94 < \left| {V_{j} } \right| < 1.06} \hfill \\ {\frac{{V_{j} - 1.06}}{1.06},} \hfill & {\left| {V_{j} } \right| > 1.06} \hfill \\ \end{array} } \right.$$

where $V_{j}$*:* Post-contingency bus voltage of bus *j*.

6 $${\text{Line Sev}}\left( {PR_{k} } \right) = \left\{ {\begin{array}{*{20}l} {PR_{k} - 1,} \hfill & {PR_{k} > 1} \hfill \\ {0,} \hfill & {PR_{k} < 1} \hfill \\ \end{array} } \right.$$

Where $PR_{k}$: Post-contingency percentage overloading of line *k*.

The two previous indices, denoted as Risk_**1**_^12^ and ***Risk***_**2**_^13^ in this study, express the main two categories of contingency indices that are dependent on quantifying the deviation of both bus voltage and line loading from the normal value without any considerations of cascading outages occurrence due to the practical corrective actions of the power grid.

### Practical operation limits

Various standards and transmission operation manuals establish distinct operational thresholds for power line operations. For instance, in^33^, the policy for overload operations is categorized into three regions, each prescribing differing strategies for corrective actions, ranging from non-cost measures to load shedding. California ISO^34^ defines System Operating Limits (SOL) encompassing:

- Normal rating (24-h rating).
- Emergency rating, further subdivided into:
- Long-term rating (4, 2, 1-h or 30-min rating).
- Short-term rating (15-min rating).

Consequently, power line flows in any given system align with four ranges: normal, long-term, short-term, and prohibited zones. Distinct operational regulations regarding corrective and preventive actions correspond to each range. The Irish grid's transmission system security and planning standard^35^ also specifies operational limits in the form of normal and emergency limits (110% of the normal limit), permitting operation within this threshold for 30 min until corrective measures are enacted.

In reference^36^, the long-term operation limit fluctuates between 1.05 and 1.8, with an operational timeframe capped at 30 min. Conversely, the short-term operation limit varies between 1.1 and 2, with a maximum operational duration of 15 min. These parameters are established by the operating utility based on thermal limits, historical data, and other aging-related factors.

**Figure 2:**
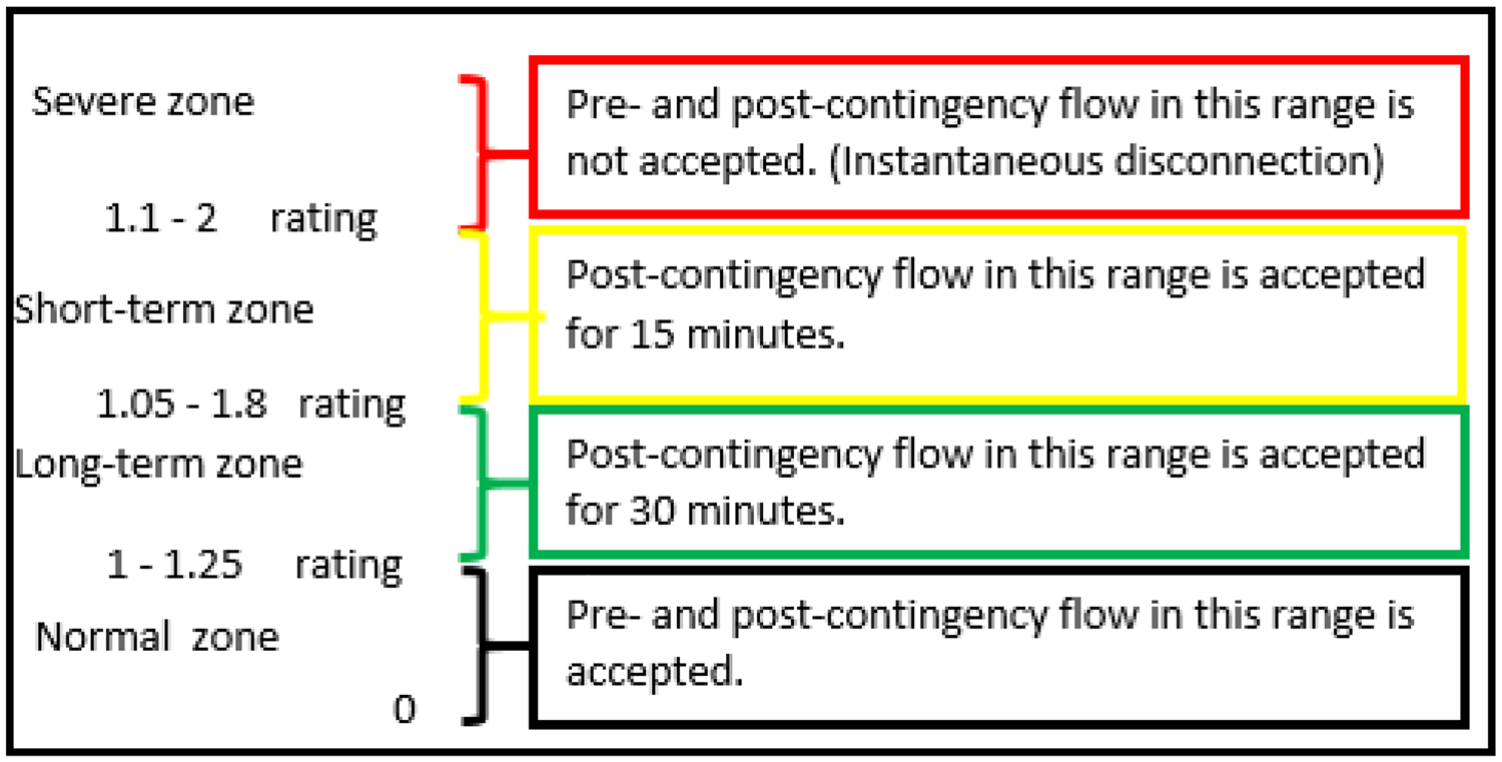
Line loading operating rules.

Post-contingency flow is admissible within any zone if corrective action can rein in the flow to below the normal rating within the stipulated timeframe. Immediate line disconnection takes place when loading surpasses the short-term limit. Limits for each operational zone exhibit substantial variability across the same grid, contingent on factors such as line manufacturing characteristics, ambient temperature, and diverse line aging influences. Figure 2 encapsulates line loading regulations as per references^35,36^.

**Figure 3:**
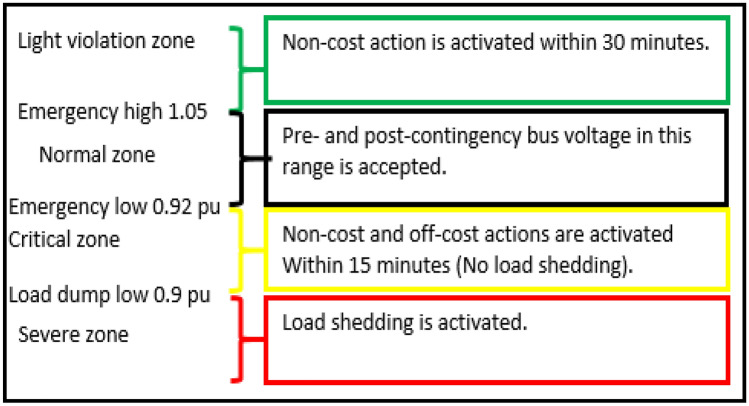
Bus voltage operating rules.

The concept of practical limits for line loading is similarly applied to bus voltage considerations. PJM Manual 03^33^ outlines appropriate corrective measures and the duration of activation for each voltage region, offering guidelines for bus voltage management. Consequently, bus voltage operation is segmented into four distinct ranges, each associated with corresponding levels of corrective actions and activation durations, as succinctly depicted in Fig. 3.

## Methodology

### New severity functions

Effective severity functions should encapsulate the impact of both contingencies and loading conditions. It should possess simplicity, facilitate the comparison of relative severity across diverse issues, and gauge the degree of deviation, given the intricate nonlinearity inherent in modern power grids.

A novel severity function for lines and bus voltages partitions the operational space into four zones: normal, light, critical, and severe. Each zone carries a specific severity weight, which corresponds to the projected corrective actions warranted for that zone. These severity weights are proposed in consideration of the allowable duration of operation before triggering corrective interventions. In essence, the suggested weights amplify as the acceptable operational time frame diminishes, and conversely, delineate the system's resilience against varying contingencies.

The proposed line/bus voltage severity function can be expressed mathematically as follows:

7 $${Sev }_{LO/V}\left({C}_{i},SC\right)={\sum }_{j=1}^{L/Npq}\sum_{k=1}^{4}{SW}_{k}*Prob\left(\frac{{S}_{j}}{{V}_{n}to}be in range k\right)$$

where *Sev*_*LO/V*_(*C*_*i*_, *SC*): The severity of *i*-th contingency under *SC* loading condition, it reflects post-contingency line overloading and bus voltage violations, *C*_*i*_: *i*-th contingency, *SC*: the system condition (demand, generation), *SW*_*k*_: The severity weight of overloading/ voltage range *k*, *L*: Number of lines in system, *N*_*pq*_: Number of load (PQ) bus in system, *Prob*(*S*_*j*_/*V*_*n*_) to be in range *k*)${\text{Prob}}({{\text{S}}}_{{\text{j}}}/{{\text{V}}}_{{\text{n}}}\mathrm{to be in range k})$ ${\text{Prob}}({{\text{S}}}_{{\text{j}}}/{{\text{V}}}_{{\text{n}}}{\text{tobeinrangek}})$: The probability of apparent power flow online *j*/Voltage of bus *n* to be in range *k*. Operation ranges and their related severity weights are selected and tabulated in Table 1.

**Table 1:** Operation ranges.

Line loading (%)	Bus voltage (p.u.)	Range (k)	severity weights
S_j_ < 100	0.92 < V_n_ < 1.05	Normal	0
100 < S_j_ < 110	V_n_ > 1.05	Light	0.2
110 < S_j_ < 120	0.9 < V_n_ < 0.92	Critical	0.5
S_j_ > 120	V_n_ < 0.9	Severe	2

### Probabilistic model of PV plant

**Figure 4:**
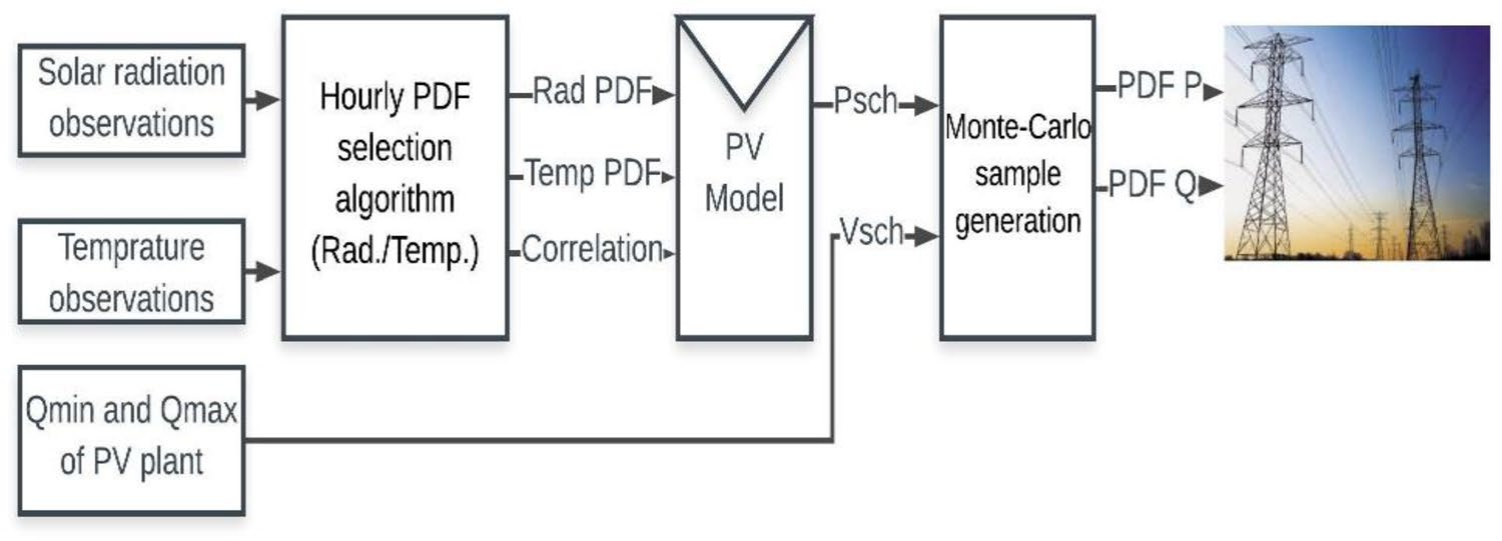
The studied probabilistic model of PV plant.

In alignment with^30^, this study establishes the optimal hourly PDF, encompassing Normal, Beta, and Weibull distributions, for both solar radiation and temperature. The applied algorithm hinges on the minimization of the root mean square error RMSE between observed solar radiation, temperature, and the optimal PDF. These observations are categorized across four seasons, each comprising 24-h data points. Consequently, the probabilistic representation of the PV system's behavior over a year encompasses 96 discrete hours. Solar radiation and temperature observations are collected at the location of the PV plant. The hourly PDF for solar radiation and temperature is established using the methodology outlined in Fig. 4.

The proposed algorithm considers all available data about both solar radiation and ambient temperature in PDF selection to give an accurate vision of PV power’s uncertainty behavior for the planning phase. On the other hand, large PV plants concerned in this study are usually connected to transmission systems. Reactive power support requirements of PV plants should be considered in the PV model, as recommended in many grid codes^37^. According to the Egyptian grid code’s recommendations^37^, external reactive power support, such as capacitor banks, is connected to large-scale PV plants that maintains the plant power factor within 0.95 Lead/Lag at rated conditions.

Recently, most PV plant models are considered small- scale plants, which are connected to distribution networks. These plants are modeled as active power sources only without any reactive power support. Therefore, a modified model for PV plants is applied by this study to satisfy grid code recommendations for large-scale ones.

After the best solar radiation and temperature PDF are estimated, PV active power behavior is estimated by Eqs. (8)–(12) as described in^38–40^, as follows:

8 $${T}_{c}={T}_{a}+{S}_{a}*({N}_{OT}-20)/0.8$$

9 $$I={S}_{a}*\left[{I}_{SC}*{K}_{i}*\left({T}_{c}-25\right)\right]$$

10 $$V={V}_{OC}-\left({K}_{v}-{T}_{c}\right)$$

11 $$FF=({V}_{MPP}*{I}_{MPP})/({V}_{OC}*{I}_{SC})$$

12 $${P}_{PV}=N*FF*V*I$$

where *T*_*C*_: The cell temperature C°, *T*_*a*_: The simulated ambient temperature C°, *S*_*a*_: The simulated solar irradiance kW/m^2^, *N*_*OT*_ :The nominal operating temperature of cell C°, *I*: The module current (A), *I*_*sc*_: The short circuit current (A), *k*_*i*_: The current temperature coefficient A/C°, *V*: The module voltage (V), V_*OC*_: The open-circuit voltage (V), *k*_V_: Voltage temperature coefficient (V/C°), *FF*: a fill factor, *V*_MPP_: The voltage at maximum power point (v), *I*_*MPP*_: Current at maximum power point (A), *P*_*PV*_: Simulated output power of the PV module, *N*: The number of modules.

Then, two equations of PV reactive power support Eqs. 13 and 14, are adapted by this research based on^41^ recommendations, as follows:

13 $${Q}_{min}=-3.122*{P}_{rated}$$

14 $${Q}_{max}=3.122*{P}_{rated}$$

where ${Q}_{min}$: The minimum reactive power supported by PV plant, ${Q}_{max}$: The maximum reactive power supported by PV plant, and *P*_rated_ :The rated power of PV plant.

### Contingency occurrence probability

Risk indices are computed as the product of the contingency's occurrence probability and its corresponding severity. The probability of a contingency's occurrence can be determined utilizing the predefined FOR. In other words, the probability of each contingency is evaluated by calculating the availability of all lines except the specific disconnected one under consideration, as delineated by the following expression^12^:

15 $${\text{Pr}}({C}_{i})={U}_{i}*\prod_{n=1,n\ne i}^{NL}(1-{U}_{n})$$

where *U*_*i*_: probability of line *i* outage or unavailability, *U*_*n*_ ${{\text{U}}}_{{\text{n}}}$ ${{\text{U}}}_{{\text{n}}}$: probability of line n outage or unavailability.

From FOR, line unavailability probability of each line can be mathematically estimated as follows:

16 $${{U}_{i}=\lambda }_{i}*{e}^{-{\lambda }_{i}*t}$$

where *λ* ${\uplambda }_{{\text{i}}}$ ${\uplambda }_{{\text{i}}}$_*i*_: The Forced outage rate (FOR) of line *i*.

**Figure 5:**
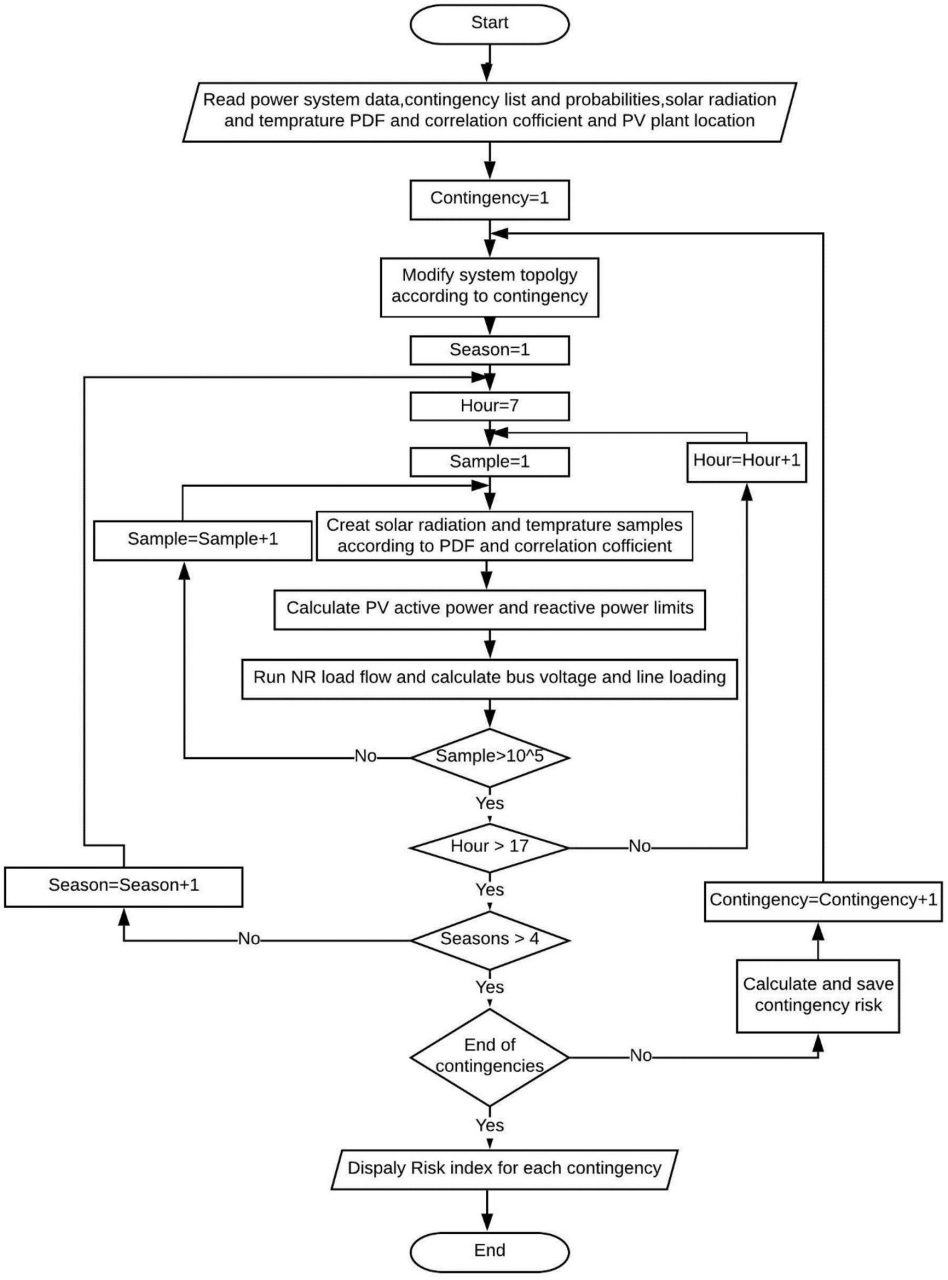
Probabilistic contingency analysis algorithm.

Upon the completion of the contingency probability assessment, the optimal PDF profiles for solar radiation and temperature are determined. Subsequently, the interrelation between temperature and radiation is quantified. Following this, the requisite reactive power support for the PV system is established. The power system's configuration is then updated to account for the specific contingency under examination. Subsequently probabilistic load flow PLF is executed to determine the probabilities of different system states. The flowchart of probabilistic contingency analysis adheres to the framework depicted in Fig. 5.

As represented in Fig. 5, for planning studies encompassing a year's duration, samples are generated to encompass four distinct seasons, with each season comprising eleven hours and utilizing a simulation of 100,000 samples per hour based on its pre-established PDF. Once the samples for system bus voltages and line flows are obtained, the Empirical Cumulative Distribution Function ECDF is approximated using curve fitting techniques to yield results. In accordance with statistical principles, the probability of a continuous random variable within a specific range is equivalent to the area under the PDF curve within that range. Accordingly, the probability can be directly derived from the Cumulative Distribution Function CDF for the predefined line overloading, and voltage operation ranges. The probability over a designated range is computed as the difference between the CDF value at the upper limit of the range and its value at the lower limit of the range. By harnessing conventional statistical methodologies, the probability term within the severity function is deduced from the ECDF as follows:

17 $$Prob({S}_{j}/{V}_{n}to be in range k)={ECDF}_{{S}_{j}/{V}_{n}}^{UL}-{ECDF}_{{S}_{j}/{V}_{n }}^{LL}$$

where ${ECDF}_{{S}_{j}/{V}_{n}}^{UL}$ , ${ECDF}_{{S}_{j}/{V}_{n}}^{LL}$: ECDF value of line *j* flow/bus *n* voltage at the upper and lower limit of range, respectively.

## Results and discussion

**Figure 6:**
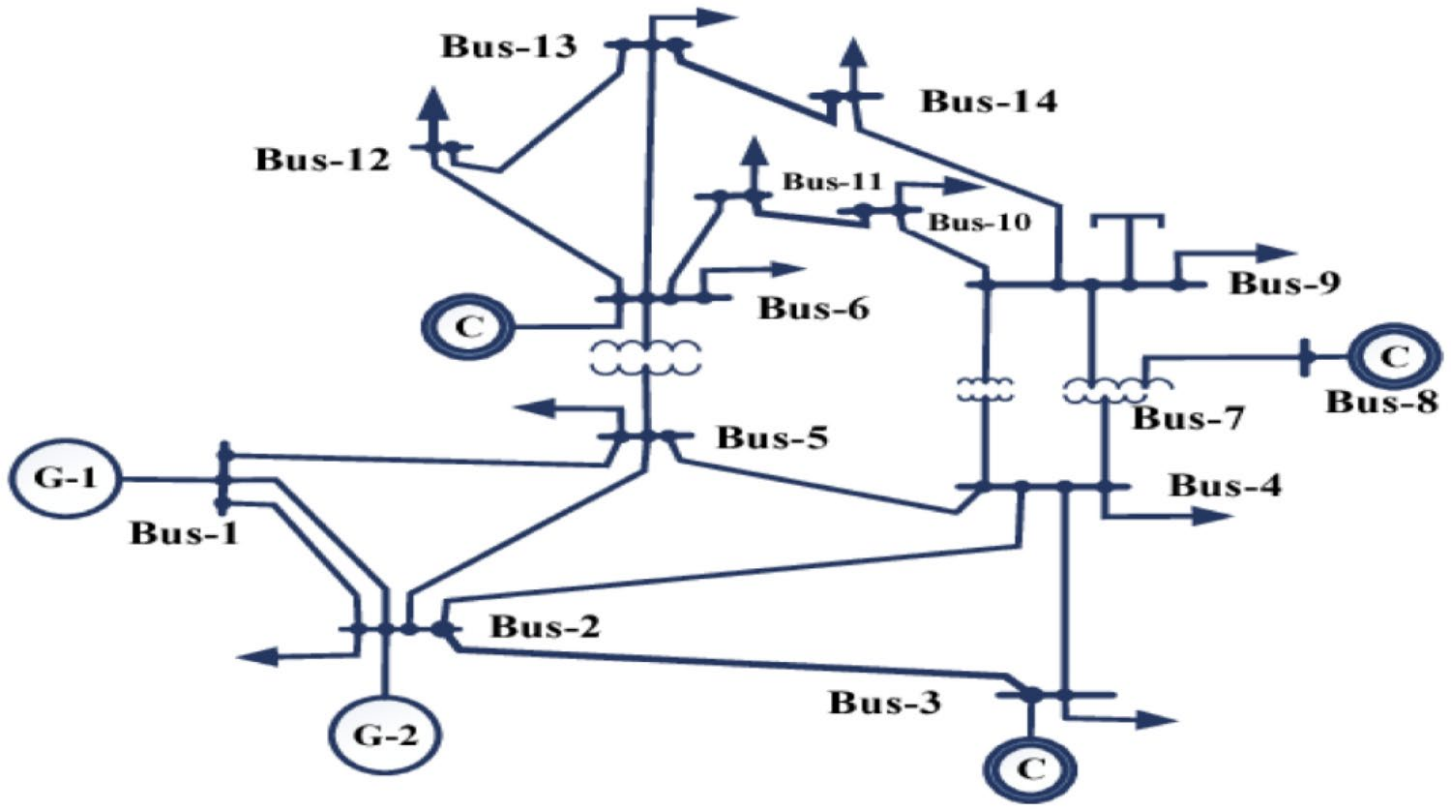
IEEE 14 bus network ^15^.

The 14-bus IEEE test system is a widely employed benchmark in power system research and analysis. It comprises 14 buses, 2 generators, 3 condensers, and 17 lines. Each bus serves as a representation of key connection points within the power network. The system incorporates three synchronous generators denoted as G1, G2, and G3, responsible for power generation and 11 loads reflecting the demand of 259 MW and 81.3 Mvar for electrical energy at various buses. Additionally, the system may include three transformers for voltage level adjustments, three shunt capacitors to provide reactive power support and enhance voltage levels, and voltage regulators to maintain desired voltage levels. IEEE 14 bus network, see Fig. 6 is studied to examine the proposed probabilistic contingency analysis.

Solar radiation and temperature observations are obtained for Benban village, Aswan, Egypt. These observations are classified according to the predefined methodology. Hourly probability distribution function best fitting is defined according to RMSE. In this study, line outage contingencies are studied. The line outage between bus 7 and bus 8 is excluded because the condenser at bus 8 will be disconnected. Therefore, it can be considered as (N-1-G) criterion. The studied contingencies’ probabilities are calculated from FOR introduced in ^41^. The new severity function is applied with the aid of the risk concept to rank contingencies. By using MATLAB language, a script is built to perform contingency analysis.

### New severity function validation

#### Line overloading severity function

**Figure 7:**
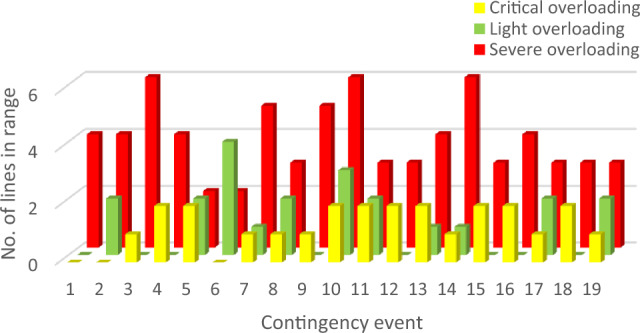
Line overloading classification.

Contingency events are simulated. The classifications of overloaded lines according to the predefined operating rules are presented in Fig. 7. As shown in Fig. 7, the total number of overloaded lines during each contingency differs significantly from 4 lines, at event 1, to 11 at overloaded lines at event 10.

In event 10, only eight lines are in the normal range from 19 available ones, with 6 lines in the severe range i.e. instantaneous disconnection. Nevertheless, this event has the least probability according to its recorded FOR, which decreases its risk. On the contrary, event 19 has only 7 violated lines, with 3 lines in severe range. But it is the most probable event, which increases its risk. Events 3 and 14 have 6 overloaded lines in the severe range with relatively high probability. Therefore, they have a high-ranking order according to the proposed risk index with a new line severity factor.

**Figure 8:**
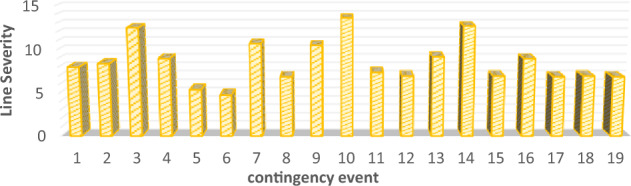
New line severity function only ranking.

**Figure 9:**
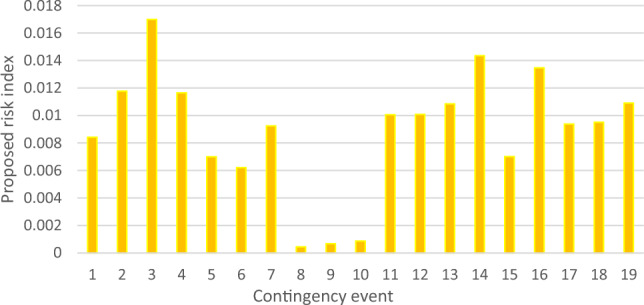
Proposed risk index ranking with line severity.

**Figure 10:**
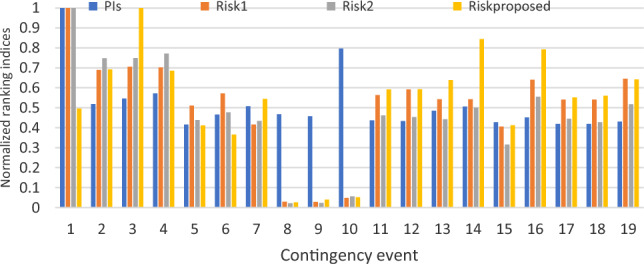
The proposed index validation.

The proposed line severity function considers the expected protective actions due to line operating rules. In addition, by using the existing risk concept, the ranking criteria will take into consideration studied events uncertainties. Therefore, the proposed risk ranking index gives a whole vision of the post-contingency grid lines state as illustrated in Figs. 8 and 9. To validate the new proposed risk index, a comparison between the proposed risk index ranking and apparent power performance index PIs introduced in^12^ and risk indices introduced in^12^ Risk1 and^13^ Risk2 respectively. The proposed risk index with new severity function and other pre-described indices are normalized around their maximum values to compare between their ranking results, as presented in Fig. 10.

PIs considers only the differences between overloaded lines and their related rating. It neglects both prospective protective actions and uncertainty of different events. Event 1 has the maximum severity according to PIs. On the other hand, according to the practical operating rules, this event has only four overloaded lines that exceed the short-term limit. In this event, only two lines (1–5) and (4–5), are highly overloaded 314% and 212% respectively. On the contrary, there are six severely overloaded lines in events 10, 3 and 14. But, it has fewer rankings than event 1.

In event 3, distributed significant overloading will occur for six lines, which may lead to partial grid collapse or even blackout. Also, this event has a relatively high probability. However, the summation of the overloading percentage is relatively less than event 1.

This miss-ranking is due to a concentrated high overloading percentage in a few lines, which appears in the case of neglecting practical protection scheme behavior and event probability.

According to Risk1 and Risk2, Event 1 is the most severe one, like the former performance index. Events 10, 8, and 9 are ranked at the end of the list due to their low probabilities.

Although these metrics consider contingency probability, line severity is still evaluated proportionally with overloading percentage. All pre-discussed indices rank different contingencies regardless of the number of violated overloaded lines or the expected protective actions. Contingency miss-ranking is repeated by these indices due to improper evaluation of different lines’ severity. For instance, event 1 is a highly ranked, the first-ranked event, according to both risk indices. While, it has less risky consequences than other events, such as 3, 10, and 14.

Unlike earlier ranking indices, the newly suggested risk index places event 3 as the most critical. Within this scenario, there are six lines that surpass the short-term limit, necessitating an immediate disconnection as operating beyond this threshold is not permissible. Moreover, this particular contingency holds a relatively elevated probability of 0.136%. The novel index accurately anticipates the state following the contingency and arranges various events based on viable and realistic operational guidelines.

#### Bus voltage severity function

**Figure 11:**
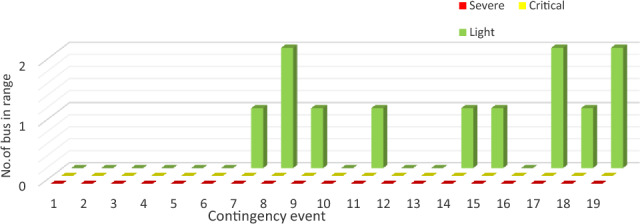
Number of bus in different ranges.

By using the same technique, the number of violated buses in voltage is calculated as shown in Fig. 11. In this study case, contingencies show bus over-voltage only. Therefore, all violated buses are lying in light range. Contingencies 9, 17 and 19 have two violated buses. While there is only one violation in contingencies 8, 10, 12, 14, 15 and 18.

The significance of contingency probability within the ranking procedure is pivotal in the context of this study, particularly when considering the uniform voltage distribution within a given range. The occurrence probability places contingency 19 in the highest position. This is attributed to its possession of the highest probability among events 9, 17, and 19. Contingency 9 is positioned at the lower end of the list, owing to its notably low probability of occurrence (0.006%), especially in comparison with contingencies 17 and 19.

**Figure 12:**
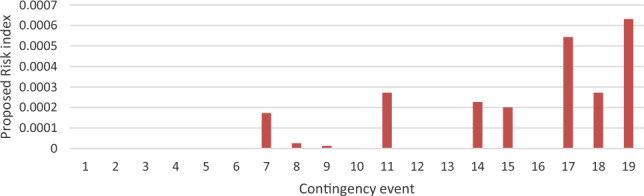
The proposed risk index ranking with bus severity.

**Figure 13:**
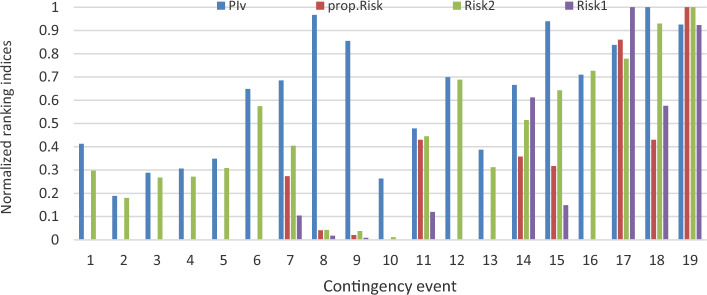
The proposed voltage risk index validation.

The arrangement of voltage severity rankings, as per the presented risk assessment, is succinctly outlined in Fig. 12. The normalized pre-mentioned indices’ ranking is compared, as presented in Fig. 13. Ranking according to PIv shows a high ranking of low probable contingencies such as 8 and 9. As it concerns only the value of bus voltage.

This index ranks contingency 18 as the most severe event. However, there is only one bus that is violated. But many buses’ voltages are higher than 1 p.u. but still in secure operating range. According to the proposed risk index, contingency 19 lies at the top of the list due to its high probability. In addition, it concerns the high number of violated buses compared to other events. The same contingency is first ranked according to Risk2, as it concerns both probability and severity like the proposed risk index.

### Probabilistic PV contingency analysis

In order to investigate the impact of a large-scale PV plant, a photovoltaic facility with 104 MWp rated capacity equivalent to 40% of the total generation capacity is incorporated into the predetermined IEEE network, connecting to various buses. To assess the coupling buses of the PV system in terms of static security, a probabilistic contingency analysis is employed within planning studies. This evaluation aims to identify coupling buses that pose lower risks. Consequently, the identical analysis is iterated across different buses, taking into account the potential for line overloading and its associated risks. Furthermore, the comparison of probabilistic contingency analyses is demonstrated across different integration buses for the PV system. This comparison aims to mitigate risks throughout the plant's operational hours over a one-year period, potentially leading to cost savings by minimizing the need for line expansion to mitigate overloading in various contingency scenarios.

#### PV coupling bus effect

**Figure 14:**
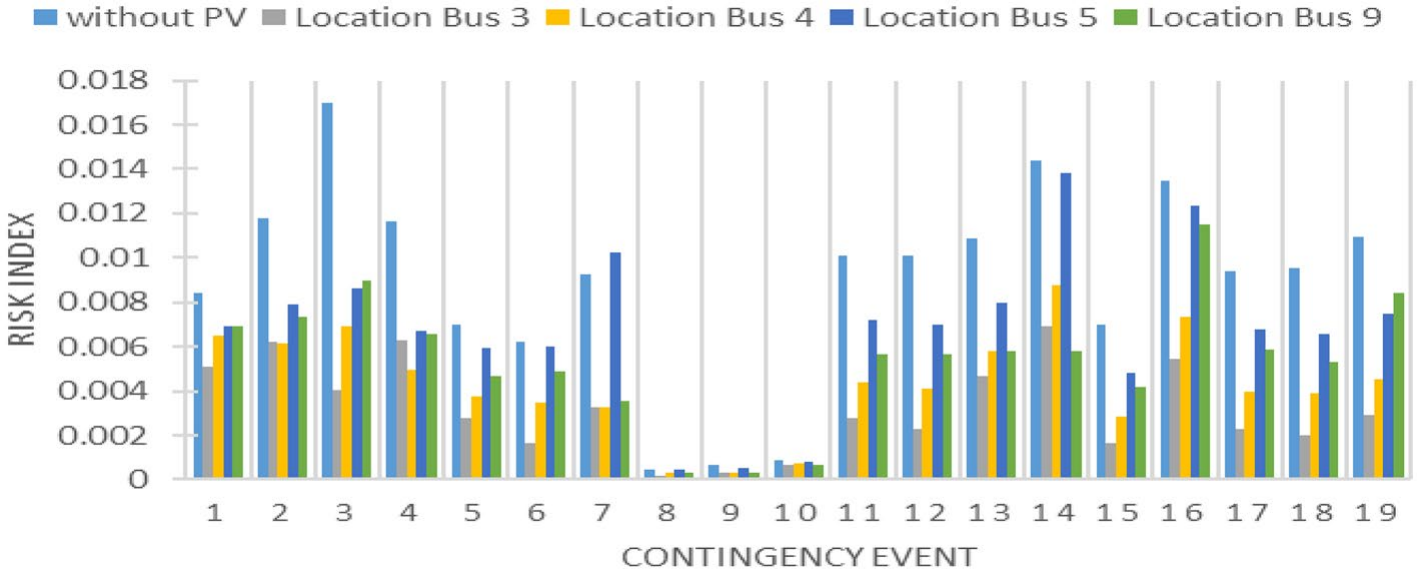
PV Line loading risk index.

**Figure 15:**
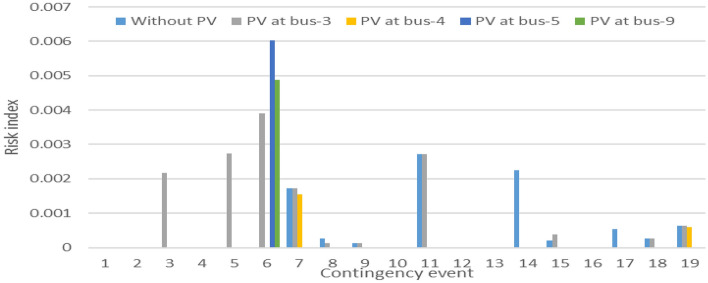
PV Bus voltage risk index.

As contingency analysis is related to both line loading and bus voltage violations, A comparison between buses (3, 4, 5, and 9) is performed for both line overloading and bus voltage violation risk consideration, as presented in Figs. 14 and 15.

As shown in Fig. 14, the integration of PV at bus 3, the heaviest loaded one, reduces the risk of line overloading significantly. Integrating the same PV plant in bus 4 is the second-best bus. As shown in Fig. 15, PV integration at bus 4 shows good results over most of the contingency lists. Although integration PV at bus 3, shows the best result in line overloading risk reduction, many bus voltage problems appeared during different contingencies for the same coupling bus. Table 2 summarizes the previous analysis of PV integration between different buses.

**Table 2:** PV generation at different buses.

	Without	Bus 3	Bus 4	Bus 5	Bus 9
Line risk					
Average	0.008889	0.003	0.004	0.007	0.005
Minimum	0.000444	0.0002	0.0003	0.0004	0.0003
Maximum	0.016995	0.007	0.009	0.01	0.01
Reduction in risk index		64%	51%	24%	39%
Voltage risk					
Average	0.0005	0.0008	0.0001	0.0003	0.0003
Minimum	0	0	0	0	0
Maximum	0.003	0.004	0.002	0.006	0.005
Reduction in risk index		− 69.47%	75.52%	31.06%	44.19%

Line expansion initiatives tend to entail higher costs in contrast to enhancements focused on reactive power. As such, it is advisable to connect the PV plant to bus 3, while also giving thought to the implementation of cost-effective reactive power support devices. In pursuit of deeper analysis, two distinct scenarios are executed to examine the effects of distributing PV capacity across diverse buses, as opposed to concentrating all capacity on a single bus. Furthermore, the replacement of conventional generation with a PV plant is subjected to examination for its potential implications.

#### Distributed PV plants

Based on the outcomes delineated in the preceding subsection, the PV plant integration at bus 3 exhibits the most favorable risk profile, followed by integration at buses 4 and 9. As a result, PV facilities possessing an equivalent 40% penetration rate and comparable solar radiation conditions are apportioned across buses 3, 4, and 9. This allocation entails simultaneous distribution in proportions of 70%, 15%, and 15%, respectively.

**Figure 16:**
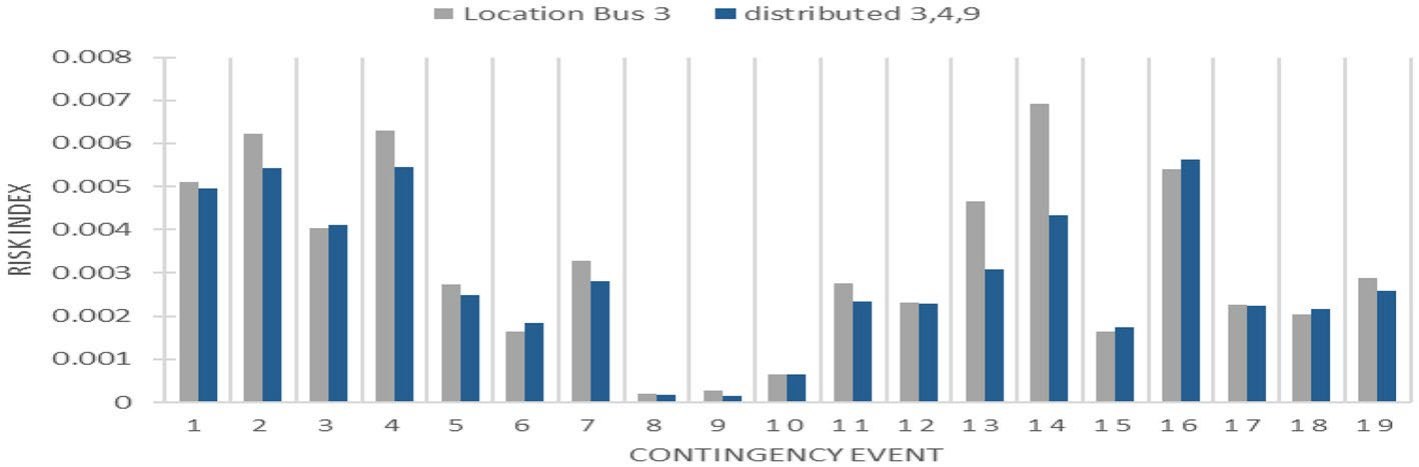
Distributed PV Line loading risk index.

**Figure 17:**
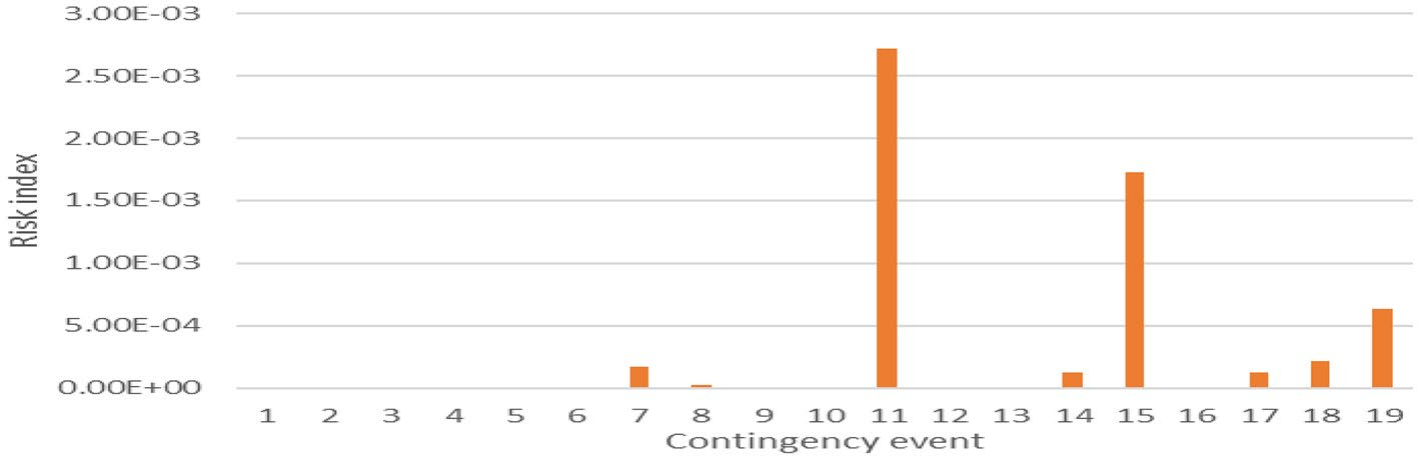
Distributed PV bus voltage risk index.

The process of probabilistic contingency analysis is then replicated for this distributed configuration without reactive power support of PV. Subsequently, a comparative assessment of the risk index is conducted between bus 3, identified as the optimal integration bus, and the distributed configuration. This comparative analysis is visually presented in Figs. 16 and 17.

Upon distributing the capacity of the PV plant across these designated buses, the risk of the line overloading demonstrates notably diminished values, surpassing even the figures observed in the optimal scenario of aggregated large-scale PV integration. Conversely, the voltage levels at all the pertinent buses remain well within acceptable limits. However, it is noteworthy that in scenarios where unity power factor generation is employed, there arise against voltage issues at the buses during contingencies. This predicament arises due to the absence of reactive power support for comparatively small-scale distributed PV installations.

#### Conventional generation replacement

In this case, conventional generation at bus 2 is totally replaced with a PV plant with the same capacity. The same contingency set is analyzed, and line overloading risk is compared with the original conventional grid case. The dispatchable power plant situated at bus 2 is substituted with a non-dispatchable PV plant.

**Figure 18:**
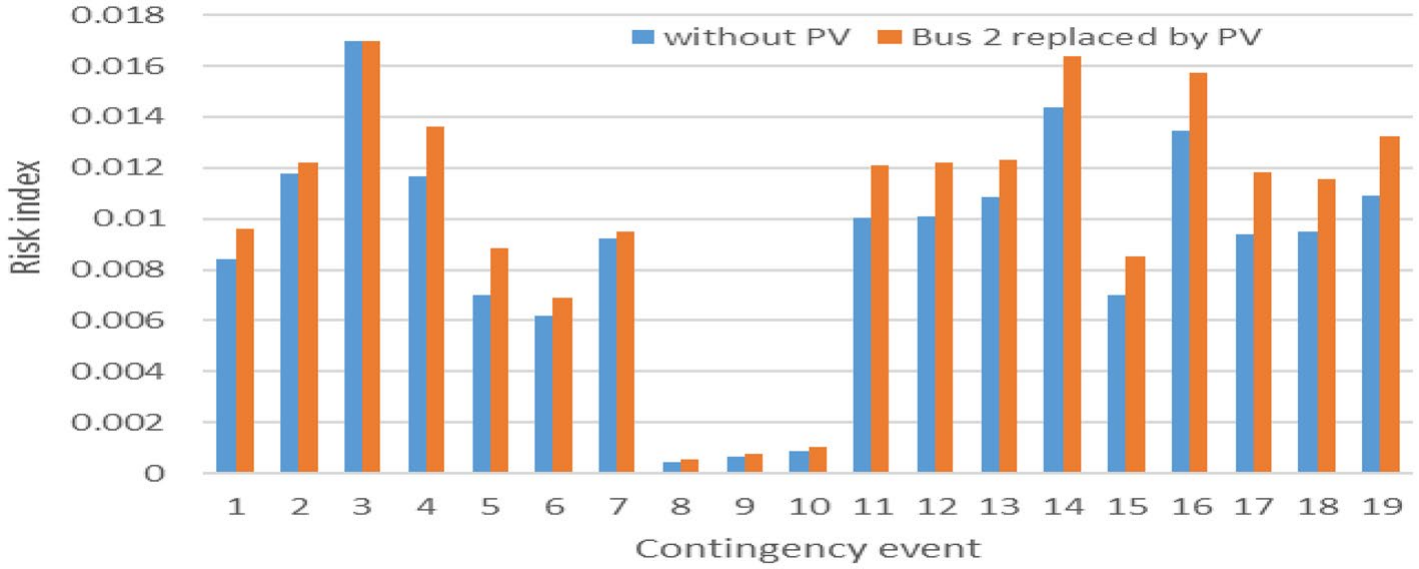
Line loading risk index, PV at bus 2.

As evident from Fig. 18, this modification results in an escalation of the risk associated with the line overloading due to the continual fluctuations in PV generation. The compensation for this fluctuation is facilitated through adjustments made at the slack bus and alterations in line flows.

**Figure 19:**
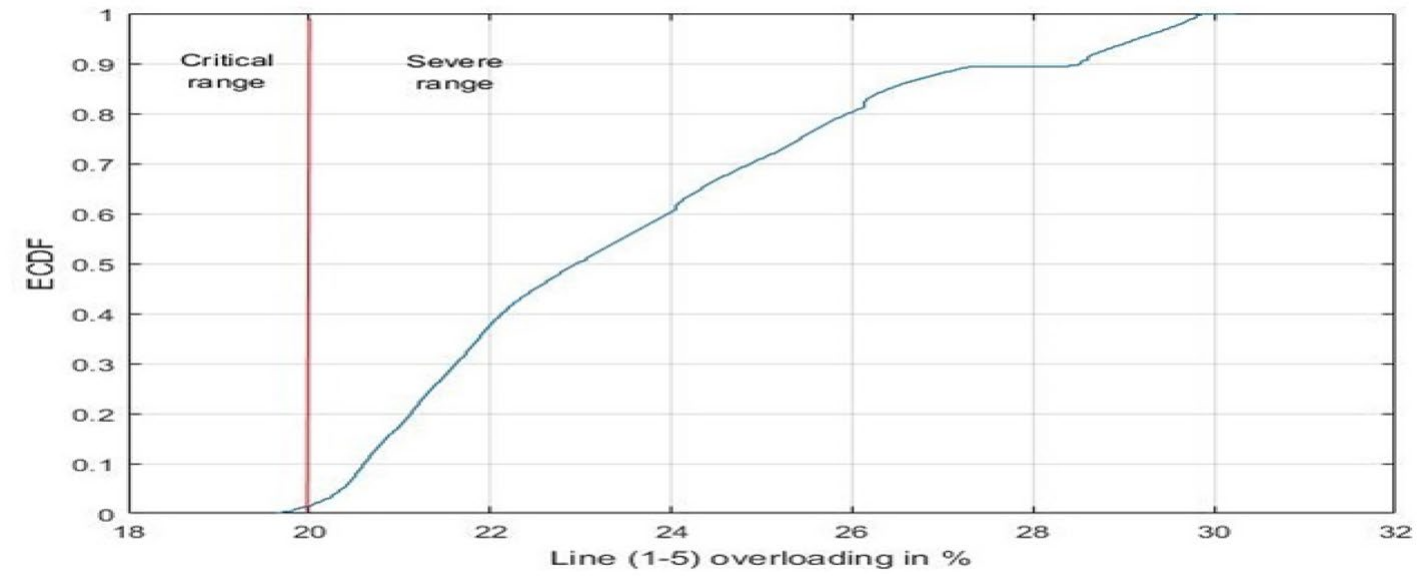
ECDF of line (1–5), contingency 17.

In the context of Contingency 17, a notable surge in overloading risk is observed, marking a 26% increase. This augmented risk is attributed to the impact on lines (1–5) and (7–9). Specifically, line (1–5) experiences an 18% overloading within the critical operational range in the original grid configuration. Notably, when the conventional generation is substituted with PV generation, as depicted in Fig. 19, the probability of transitioning into the severe operational range rises significantly to 98%. This heightened probability translates to an instantaneous outage scenario.

**Figure 20:**
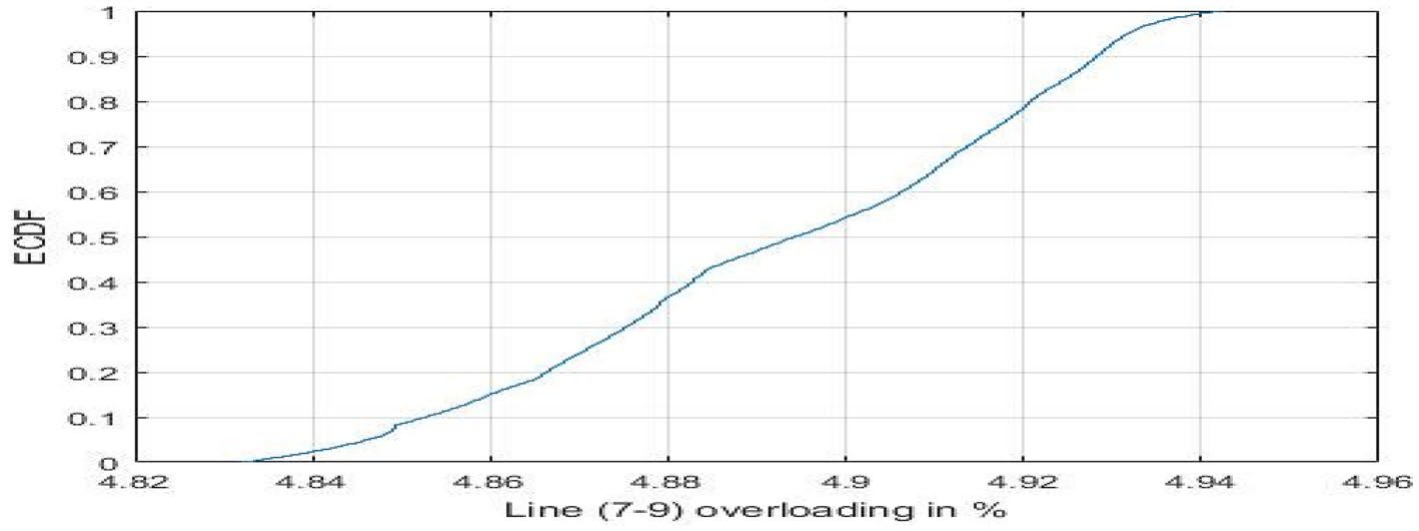
ECDF of line (7–9), contingency 17.

Furthermore, line (7–9) initially operates within the normal range within the conventional grid setup. However, upon replacing conventional power with PV power, its operational status shifts to the light overloading range, as illustrated in Fig. 20.

The substitution of conventional generation with photovoltaic (PV) sources in power grids introduces adverse effects on contingency analysis, as evidenced by the findings in Fig. 18. This modification amplifies the risk of line overloading due to persistent fluctuations in PV generation. To manage these fluctuations, adjustments at the slack bus and alterations in line flows become necessary. In the specific context of Contingency 17, a substantial increase in overloading risk is evident, with a notable 26% surge. This heightened risk is particularly attributed to the impact on lines (1–5) and (7–9). Line (1–5) experiences an 18% overloading within the critical operational range in the original grid configuration. Strikingly, when conventional generation is replaced by PV generation, as depicted in Fig. 19, the probability of transitioning into the severe operational range spikes significantly to 98%. This elevated probability implies a heightened susceptibility to instantaneous outage scenarios. Additionally, line (7–9), which initially operates within the normal range in the conventional grid setup, shifts to the light overloading range upon the substitution of conventional power with PV power, as illustrated in Fig. 20. These observations underscore the need for careful consideration of the impacts of transitioning from conventional to PV generation in power grid planning and contingency analysis.

## Conclusion

In the realm of power system operations and planning, the intricate task of contingency analysis assumes paramount significance. The proliferation of stochastic PV plants in modern grids introduces a heightened level of uncertainty into the analysis landscape. In light of this, a shift from deterministic to probabilistic analysis emerges as imperative to effectively address the variability inherent in uncertain behaviors. Traditional contingency ranking metrics, centered on deviations between observed line loadings and bus voltages and their permissible thresholds, are juxtaposed with practical corrective actions categorized into light, critical, and severe zones.

This study presents a novel severity function framework that not only accounts for anticipated grid remedial actions but also classifies line and bus voltages according to the temporal window available before enacting remedial measures. Incorporating predefined weighting factors, this approach discriminates between overloaded lines and buses, yielding a marked enhancement in contingency ranking over conventional indices. To encompass both contingency incidence and PV generation unpredictability, a probabilistic contingency analysis paradigm is proposed, underpinned by operational guidelines for power system elements, encompassing both traditional and PV-driven scenarios.

Leveraging historical data, a framework for quantifying contingency occurrence uncertainty is fashioned, while the PV generation uncertainty is harnessed through the utilization of the PLF algorithm in conjunction with established contingency analysis tools. A probabilistic model is crafted for PV plants, grounded in historical solar radiation and temperature data, culminating in the derivation of hourly probability density functions. Inclusive of diverse grid code requisites for PV plant integration, this model encompasses reactive power demands.

Verification on IEEE 14-bus network elucidates the efficacy of probabilistic contingency analysis in discerning risks linked to line overloading and bus voltage. While PV expansion does yield partial line loading improvement compared to conventional methods, its inherent variability limits its transformative impact. The study delineates a strategic comparison of risk indices across various PV integration sites, culminating in the identification of optimal integration buses. Furthermore, juxtaposing concentrated PV capacity with distributed scenarios underscores the superiority of shared capacity distribution in minimizing line overloading risks. The investigation's exclusion of reactive power support underscores a pronounced decline in voltage risk, spotlighting the indispensable role of reactive power support.

Evidencing the merit of intensive probabilistic security evaluations for PV plant integration, this research offers indispensable insights for coupling bus selection, thereby maximizing the grid's technical advantages from a security standpoint. Future work may involve the development and validation of contingency analysis tools specifically tailored for assessing the resilience of power systems to combined uncertainties from high-penetration wind and PV integration, providing realistic insights for grid operators and planners.

## Data Availability

The datasets analyzed during the current study are available in the [Renewable. Ninja] repository, [https://www.renewables.ninja/]. The datasets generated during the current study available from the corresponding author on reasonable request.
